# The β-cell GHSR and downstream cAMP/TRPM2 signaling account for insulinostatic and glycemic effects of ghrelin

**DOI:** 10.1038/srep14041

**Published:** 2015-09-15

**Authors:** Tomoyuki Kurashina, Katsuya Dezaki, Masashi Yoshida, Rauza Sukma Rita, Kiyonori Ito, Masanobu Taguchi, Rina Miura, Makoto Tominaga, Shun Ishibashi, Masafumi Kakei, Toshihiko Yada

**Affiliations:** 1 Division of Integrative Physiology, Department of Physiology, Jichi Medical University School of Medicine, Yakushiji 3311-1, Shimotsuke, Tochigi 329-0498, Japan; 2 Department of Internal Medicine, Saitama Medical Center, Jichi Medical University School of Medicine, Omiya 1-847, Saitama 337-8503, Japan; 3 Division of Cell Signaling, Okazaki Institute for Integrative Bioscience, National Institute for Physiological Sciences, Okazaki, Aichi 444-8787, Japan; 4 Division of Endocrinology and Metabolism, Department of Internal Medicine, Jichi Medical University School of Medicine, Yakushiji 3311-1, Shimotsuke, Tochigi 329-0498, Japan; 5 Department of Development Physiology, Division of Adaptation Development, National Institute for Physiological Sciences, Okazaki, Aichi 444-8585, Japan

## Abstract

Gastric hormone ghrelin regulates insulin secretion, as well as growth hormone release, feeding behavior and adiposity. Ghrelin is known to exert its biological actions by interacting with the growth hormone secretagogue-receptor (GHSR) coupled to G_q/11_-protein signaling. By contrast, ghrelin acts on pancreatic islet β-cells via G_i_-protein-mediated signaling. These observations raise a question whether the ghrelin action on islet β-cells involves atypical GHSR and/or distinct signal transduction. Furthermore, the role of the β-cell GHSR in the systemic glycemic effect of ghrelin still remains to be defined. To address these issues, the present study employed the global GHSR-null mice and those re-expressing GHSR selectively in β-cells. We here report that ghrelin attenuates glucose-induced insulin release via direct interaction with ordinary GHSR that is uniquely coupled to novel cAMP/TRPM2 signaling in β-cells, and that this β-cell GHSR with unique insulinostatic signaling largely accounts for the systemic effects of ghrelin on circulating glucose and insulin levels. The novel β-cell specific GHSR-cAMP/TRPM2 signaling provides a potential therapeutic target for the treatment of type 2 diabetes.

Ghrelin, an acylated 28-amino acid peptide produced predominantly in the stomach^1^, was discovered as the endogenous ligand for a G-protein coupled receptor (GPCR), growth hormone (GH) secretagogue-receptor (GHSR)^2^, which is widely expressed throughout the body^3^. Ghrelin promotes GH release, feeding behavior and adiposity^1^,^4^,^5^. GHSR-null mice are refractory to ghrelin’s stimulation of GH release and appetite, confirming GHSR as the specific ghrelin receptor for these actions^6^. Ghrelin also inhibits glucose-stimulated insulin secretion from perfused pancreas, isolated islets and β-cell lines^7^,^8^,^9^,^10^,^11^. Ghrelin and GHSR are located in the pancreatic islets^3^,^12^. It is currently thought that biological actions of ghrelin are mediated by GHSR, which is coupled primarily to the G_q/11_-phospholipase C signaling^2^. In contrast, we have found that the insulinostatic action of ghrelin are produced via pertussis toxin (PTX)-sensitive G-protein Gα_i2_ in β-cells, which leads to attenuation of cAMP and [Ca^2+^]_i_ signaling in β-cells and insulin release from islets^13^,^14^. However, the molecular identity of the receptor that is coupled to G_i_ for insulinostatic ghrelin action in β-cells remains to be defined. Presence of unidentified ghrelin receptor has been suggested by the observation that ghrelin exerts some effects in the cells and tissues that do not express GHSR^15^,^16^,^17^.

*In vivo* analysis revealed that administration of ghrelin attenuates insulin release and impairs glucose tolerance in rodents and humans^7^,^11^,^18^,^19^. Ghrelin transgenic mice with increased circulating ghrelin exhibited deteriorated glucose tolerance without change in blood glucose levels during insulin tolerance tests (ITT)^20^. Conversely, administration of ghrelin antagonists^7^,^21^,^22^ and inhibitor^23^ of ghrelin *O*-acyltransferase (GOAT), the enzyme that acylates the third serine residue of ghrelin^24^,^25^, enhances insulin secretory responses and lowers blood glucose concentrations during glucose tolerance tests (GTT). In consistent with the pharmacological studies, mice lacking gene of ghrelin^21^ and those of GOAT^26^ showed improved glucose tolerance and enhanced plasma insulin release under normal chow condition. Furthermore, in these ghrelin-knockout (KO) mice and GOAT-KO mice, high-fat diet-induced glucose intolerance was prevented due to enhanced insulin secretory response to glucose. Ablation of ghrelin also improved glucose tolerance and enhances insulin secretion in leptin-deficient ob/ob mice^27^. These findings suggest that the insulinostatic function of ghrelin would affect blood glucose levels, and manipulation of the β-cell ghrelin action could provide a novel tool to optimize insulin release for achieving normoglycemia. Thus, KO studies on the ligand ghrelin have been substantially conducted. In contrast, GHSR-KO mice have been little analyzed for their insulin releasing ability and glucose metabolism, although they reportedly show improved insulin sensitivity when fed a high-fat diet^28^,^29^. In a gain-of-function study, ghrelin was reported to induce peripheral insulin resistance in humans^30^ and glucose output from porcine hepatocytes^31^. Thus, whether GHSR, particularly that expressed in β-cells, is implicated in ghrelin’s glycemic effect is not fully understood.

In this study, we aimed to clarify whether insulinostatic ghrelin action is mediated by ordinary GHSR in islet β-cells by using GHSR-null mice. Furthermore, the study explored the role of the β-cell GHSR in the glycemic effects of ghrelin administration and endogenous ghrelin as assessed by the effect of ghrelin antagonists.

## Results

### Ghrelin attenuates glucose-induced insulin release in a GHSR-dependent manner in mouse islets

**Figure 1:**
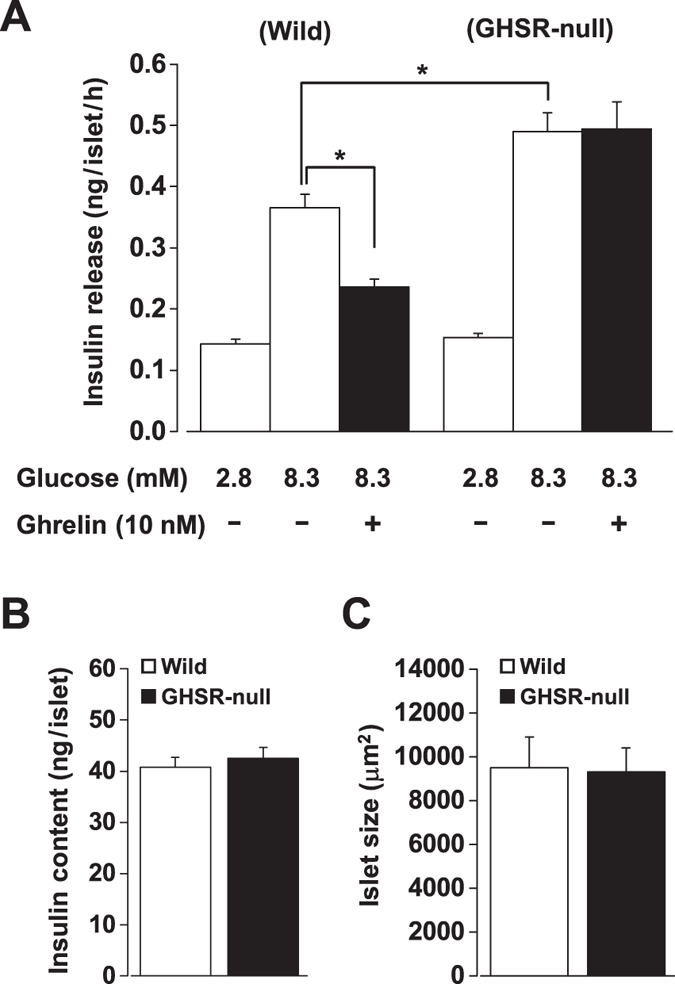
Exogenous ghrelin and endogenous islet-derived ghrelin attenuate glucose-induced insulin release in a GHSR-dependent manner in mouse islets. (**A**) Glucose (8.3 mM)-induced insulin release in isolated islets was inhibited by exogenous ghrelin (10 nM) in wild-type mice. In isolated islets from GHSR-null mice, ghrelin (1  nM) failed to attenuate glucose (8.3 mM)-induced insulin release. The glucose (8.3 mM)-induced insulin release *per se* was larger in GHSR-null mice than wild-type mice (*n* = 8–12 tubes of batch incubation). *P < 0.05. (**B**) Insulin content per islet (*n* = 8 tubes) and (**C**) mean islet size (*n* = 79–107 islets) were identical between wild-type and GHSR-null mice.

In wild-type mice, insulin release from isolated islets under static batch-incubation was stimulated by 8.3 mM glucose compared to 2.8 mM glucose (*P* < 0.01, Fig. 1A). The glucose (8.3 mM)-induced insulin release was inhibited by exogenous ghrelin (Fig. 1A), as reported^7^. In isolated islets from GHSR-null mice, in contrast, ghrelin (10 nM) failed to attenuate the glucose (8.3 mM)-induced insulin release (Fig. 1A). Furthermore, the glucose (8.3 mM)-induced insulin release *per se* was significantly larger in GHSR-null mice than wild-type mice, while basal levels of insulin release at 2.8 mM glucose were not different (Fig. 1A). Insulin content per islet and islet size were identical between wild-type and GHSR-null mice (Fig. 1B,C), suggesting an equal β-cell masses. These data indicate that both exogenous ghrelin and endogenous islet-derived ghrelin attenuate glucose-induced insulin release in a GHSR-dependent manner.

### Ghrelin inhibits glucose-induced cAMP production via GHSR in mouse islets

**Figure 2:**
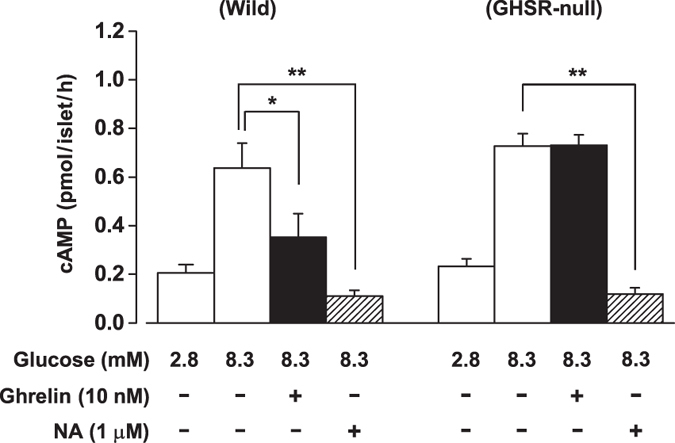
Ghrelin inhibits glucose-induced cAMP production via GHSR in mouse islets. In the presence of PDE inhibitor IBMX (500 μM), glucose (8.3 mM)-induced cAMP production in isolated islets from wild-type mice was inhibited by ghrelin (10 nM). Ghrelin failed to suppress 8.3 mM glucose-induced cAMP production in isolated islets from GHSR-null mice. Noradrenaline (NA) (1 μM) strongly suppressed the cAMP production in both groups of islets (*n* = 6 tubes of batch incubation). *P < 0.05; **P < 0.01.

In the presence of phosphodiesterase (PDE) inhibitor IBMX (500 μM), 8.3 mM glucose stimulated cAMP production in islets under static incubation, compared to 2.8 mM glucose (*P* < 0.05) (Fig. 2). The glucose (8.3 mM)-induced cAMP increase was significantly inhibited by application of ghrelin (10 nM) (Fig. 2). In islets isolated from GHSR-null mice, ghrelin (10 nM) failed to suppress the 8.3 mM glucose-induced cAMP production, demonstrating that ghrelin inhibits cAMP production via interacting with GHSR. In contrast, noradrenaline (1 μM) strongly suppressed cAMP production in islets of both groups (Fig. 2), indicating that α2-adrenergic receptor-mediated G_i/o_ signaling is intact in the islets of GHSR-null mice.

### Ghrelin attenuates glucose-induced [Ca^2+^]_i_ increases in β-cells in a GHSR-dependent manner

**Figure 3:**
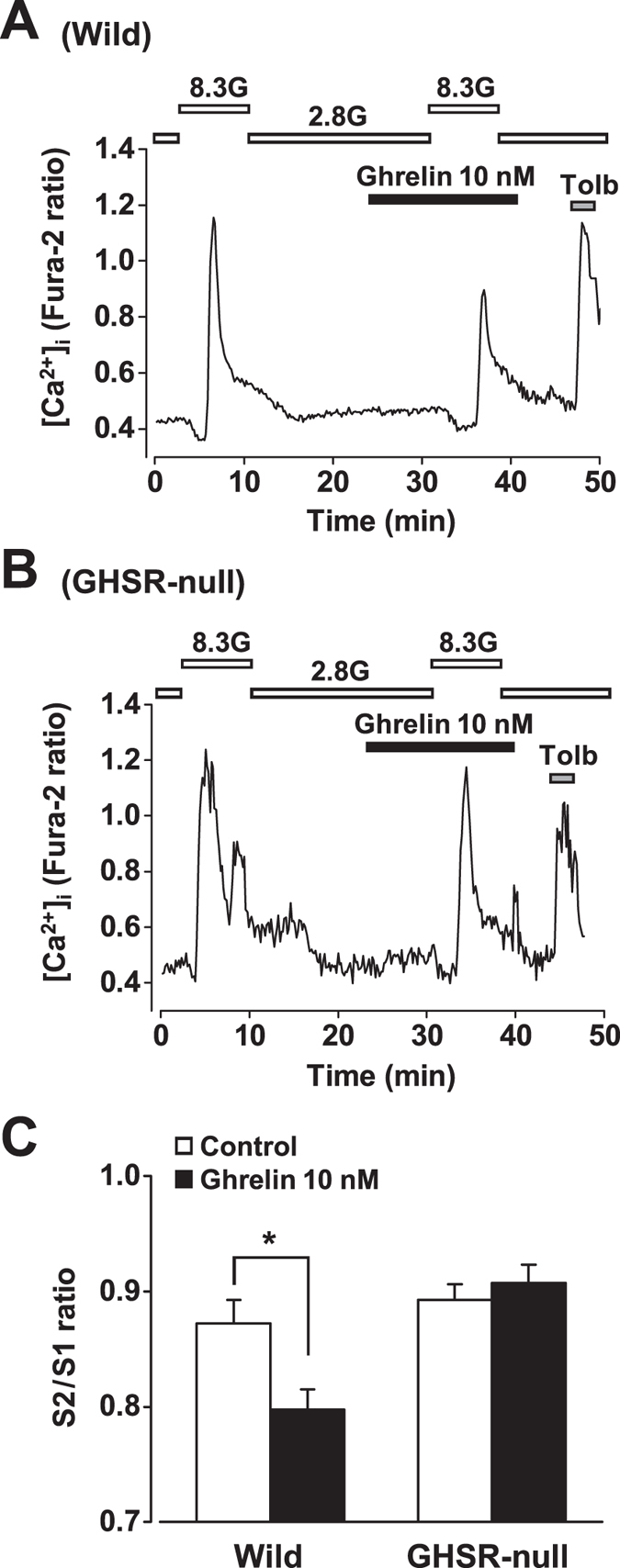
Ghrelin attenuates glucose-induced [Ca^2+^]_i_ increases in β-cells in a GHSR-dependent manner. (**A**) Glucose (8.3 mM) increased [Ca^2+^]_i_ in β-cells isolated from islets of wild-type mice. The glucose (8.3 mM)-induced [Ca^2+^]_i_ increase was suppressed by ghrelin (10 nM), which was added to superfusion solution 5 min prior to the second glucose stimulation. (**B**) Ghrelin failed to suppress glucose (8.3 mM)-induced [Ca^2+^]_i_ increases in β-cells from GHSR-null mice. (**C**) The ratio of the peak amplitude of [Ca^2+^]_i_ increases in responses to the second glucose (8.3 mM) stimulation (S2) over that to the first glucose stimulation (S1), S2/S1, was decreased by ghrelin (10 nM), indicating inhibition of [Ca^2+^]_i_ responses. The ability of ghrelin to suppress glucose (8.3 mM)-induced [Ca^2+^]_i_ increases was abolished in GHSR-null β-cells (*n* = 74–150 cells). *P < 0.05.

A repetitive stimulation with 8.3 mM glucose induced repeated [Ca^2+^]_i_ increases in mouse single β-cells. The ratio of the peak amplitude of [Ca^2+^]_i_ response to the second glucose stimulation (S2) to that to the first stimulation (S1), S2/S1, was 0.87 ± 0.02 (n = 120) in control wild-type β-cells. Ghrelin (10 nM), added to superfusion solution 5 min prior to the second glucose stimulation, suppressed [Ca^2+^]_i_ responses to 8.3 mM glucose, decreasing S2/S1 ratio to 0.79 ± 0.02 (*P* < 0.05, n = 150) (Fig. 3A,C). In GHSR-null mice, by contrast, ghrelin failed to suppress the glucose-induced [Ca^2+^]_i_ increases in β-cells (Fig. 3B): S2/S1 ratio was unaltered by ghrelin (control; 0.89 ± 0.01, n = 74 vs. ghrelin; 0.91 ± 0.02, n = 107) (Fig. 3C).

### Ghrelin attenuates glucose-induced TRPM2 activation in a GHSR-dependent manner

**Figure 4:**
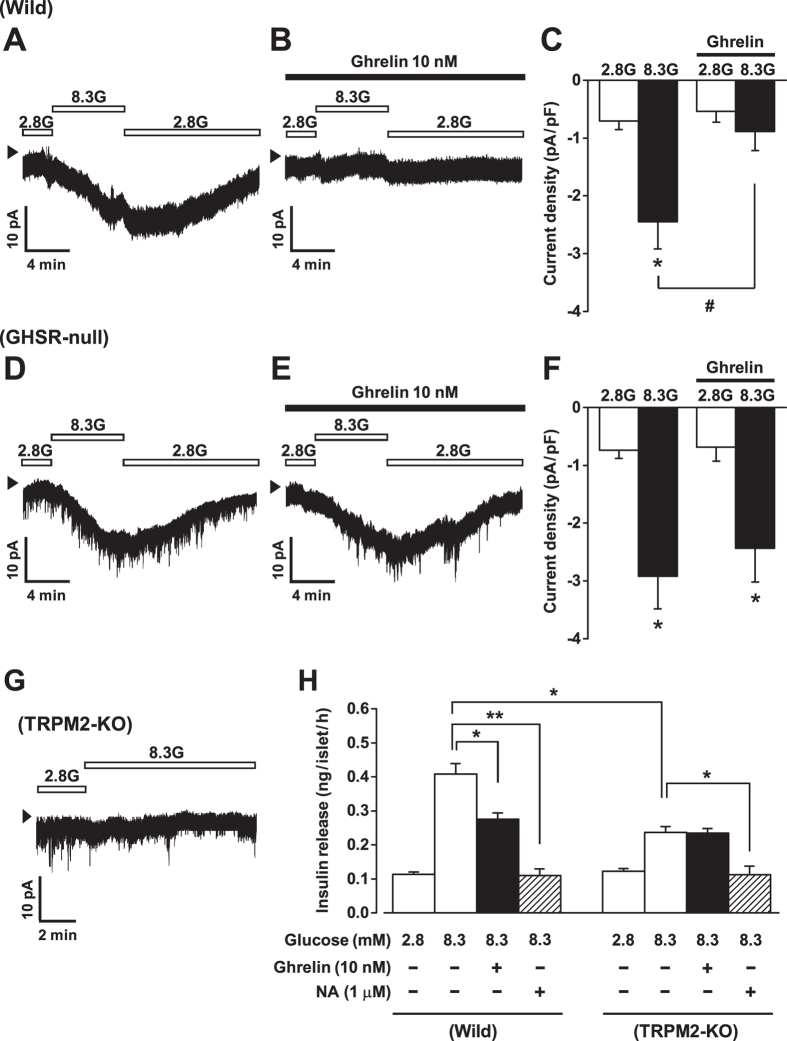
Ghrelin attenuates glucose-induced TRPM2 activation in a GHSR-dependent manner. (**A**) At a holding potential of −70 mV, an increase in external glucose concentration from 2.8 mM (2.8G) to 8.3 mM (8.3G) increased inward TRPM2 current in wild-type β-cells in a reversible manner. (**B**) Ghrelin (10 nM) attenuated the 8.3G-elicited current in wild-type β-cells. (**C**) The 8.3 mM glucose-induced increase in inward current density was significantly attenuated by ghrelin in wild-type β-cells. (**D–F**) In β-cells from GHSR-null mice, the TRPM2 currents were increased by 8.3G to a similar extent to those in wild-type β-cells, but the currents increased by 8.3G were not altered by ghrelin. (**G**) The glucose (8.3 mM) did not induce NSCC current in β-cells from TRPM2-KO mice. *n* = 5–8 (single β-cells). *P < 0.05 vs. 2.8G; ^#^*P* < 0.05 vs. 8.3G alone in wild-type. Arrow heads indicate zero current level. (**H**) The glucose (8.3 mM)-induced insulin release was significantly lower in isolated islets from TRPM2-KO mice than that from wild-type mice, while basal insulin release at 2.8 mM glucose was unchanged. Ghrelin failed to attenuate glucose (8.3 mM)-induced insulin release in the TRPM2-KO islets, while noradrenaline (NA) inhibited it (*n* = 8–12 tubes of batch incubation). *P < 0.05, **P < 0.01.

The non-selective cation channel (NSCC) currents in mouse β-cells under amphotericin B-perforated whole-cell clamp were measured in the presence of 100 μM tolbutamide to inhibit the K_ATP_ channel and thereby exclude involvement of this channel in the currents. At a holding potential of −70 mV, an increase in external glucose concentration from 2.8 mM to 8.3 mM increased the NSCC current in wild-type β-cells in a reversible manner (Fig. 4A). Ghrelin markedly decreased the 8.3 mM glucose-elicited current densities to −0.89 ± 0.33 pA/pF from −2.45 ± 0.47 pA/pF (*P* < 0.05, n = 5) (Fig. 4B,C). In β-cells from GHSR-null mice, the NSCC currents were increased by 8.3 mM glucose (Fig. 4D) to a level similar to that in wild-type β-cells (Fig. 4C,F) but, notably, the 8.3 mM glucose-induced currents were not altered by ghrelin (Fig. 4E,F).

Previous study^32^ demonstrated that this glucose-induced NSCC current is inhibited by 2-APB, a blocker of transient receptor potential melastatin 2 (TRPM2) channel, and is not elicited in TRPM2-deficient β-cells, indicating that the current is passing through TRPM2 channels. As confirmed in Fig. 4G, glucose (8.3 mM) failed to induce NSCC current in β-cells from TRPM2-KO mice. We examined whether the ghrelin-induced attenuation of TRPM2 currents is involved in the ghrelin action to suppress insulin release. The glucose (8.3 mM)-induced insulin release was significantly lower in isolated islets from TRPM2-KO mice than that from wild-type mice, while basal insulin release at 2.8 mM glucose was unchanged (Fig. 4H). Moreover, ghrelin failed to attenuate glucose (8.3 mM)-induced insulin release in the TRPM2-KO islets (Fig. 4H). In contrast, noradrenaline (1 μM) inhibited glucose-induced insulin release in islets from TRPM2-KO, as well as wild-type, mice (Fig. 4H). These results suggest that the action of ghrelin to inhibit TRPM2 channel conductance is causally implicated in its insulinostatic action.

### Specific re-expression of GHSR in β-cells of GHSR-null/Ins-Cre mice

**Figure 5:**
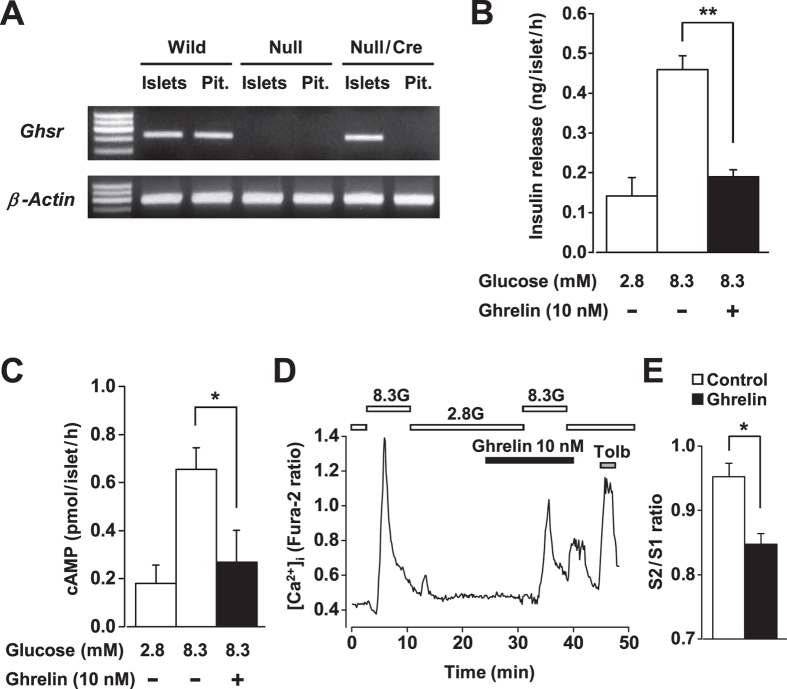
Specific re-expression of GHSR in β-cells of GHSR-null/Ins-Cre mice. (**A**) RT-PCR analysis showed that the Ghsr mRNA was expressed in isolated islets and pituitary (Pit.) from wild-type mice (Wild), but not detectable in those from GHSR-null mice (Null). GHSR-null/Ins-Cre mice (Null/Cre) exhibited Ghsr mRNA expression in islets but not in pituitary. (**B**) In isolated islets from GHSR-null/Ins-Cre mice, ghrelin (10 nM) markedly attenuated the glucose (8.3 mM)-induced insulin release. *n* = 8 (tubes of batch incubation). (**C**) In the presence of IBMX (500 μM), glucose (8.3 mM)-induced cAMP production in isolated islets from GHSR-null/Ins-Cre mice was inhibited by ghrelin (*n* = 6). (**D** and **E**) In single β-cells from GHSR-null/Ins-Cre mice, ghrelin suppressed glucose (8.3 mM)-induced [Ca^2+^]_i_ increases. *n* = 148–214 (single β-cells). *P < 0.05, **P < 0.01.

In order to specifically re-express GHSR in β-cells of GHSR-null mice, GHSR-null mice were bred with rat insulin promoter-driven Cre recombinase (Ins-Cre) mice to obtain GHSR-null/Ins-Cre mice. RT-PCR analysis showed that the Ghsr mRNA was expressed in isolated islets and pituitary from wild-type mice, but not detectable in those from GHSR-null mice. As expected, GHSR-null/Ins-Cre mice exhibited Ghsr mRNA expression in islets but not in pituitary, indicating that GHSR expression was specifically restored in islets of the null mice by bred with Ins-Cre mice (Fig. 5A). Functional re-expression of GHSR was also examined. In isolated islets from GHSR-null/Ins-Cre mice, ghrelin (10 nM) markedly attenuated the glucose (8.3 mM)-induced insulin release (Fig. 5B) and cAMP production (Fig. 5C). In single β-cells from GHSR-null/Ins-Cre mice, ghrelin significantly suppressed glucose (8.3 mM)-induced [Ca^2+^]_i_ increases (Fig. 5D,E), in which the suppression of response amplitude was identical to that in wild-type β-cells (Fig. 3A,C). These data demonstrated that re-expression of GHSR renders β-cells responsive to ghrelin and thereby restores its insulinostatic signaling.

### Endogenous and exogenous ghrelin regulate systemic blood glucose and plasma insulin levels via β-cell GHSR

**Figure 6:**
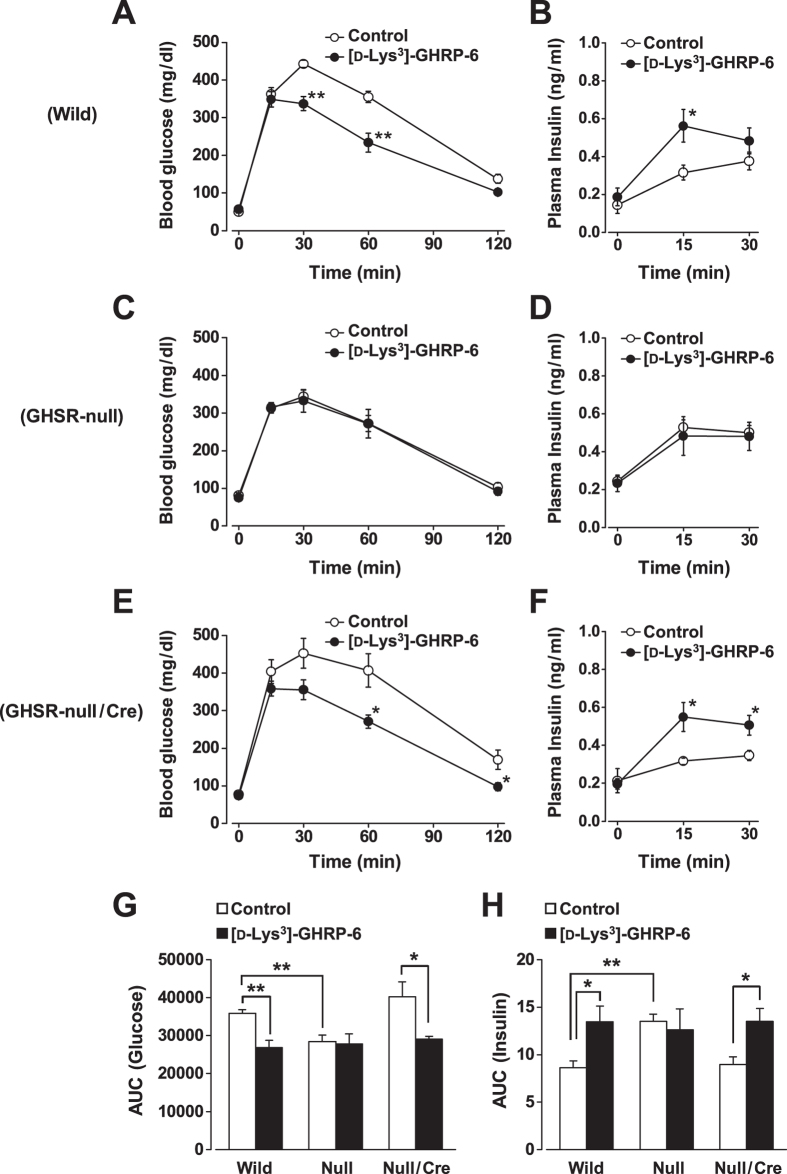
Endogenous ghrelin regulates systemic blood glucose and plasma insulin levels via β-cell GHSR. (**A**) In glucose tolerance tests (GTT), when a ghrelin antagonist [D-Lys^3^]-GHRP-6 (1 μmol/kg, i.p.) was injected simultaneously with 2 g/kg glucose into the wild-type mice, increases in plasma glucose were attenuated and (**B**) the insulin response was enhanced compared to saline-injected mice. (**C**,**D**) The GHSR-null mice exhibited smaller blood glucose increases and larger plasma insulin responses in GTT than wild-type mice. Moreover, administration of [D-Lys^3^]-GHRP-6 failed to affect blood glucose and plasma insulin responses in GTT in GHSR-null mice. (**E**,**F**) In GHSR-null/Ins-Cre mice, the glucose and insulin curves in GTT were similar to those in wild-type mice. Administration of [D-Lys^3^]-GHRP-6 was able to attenuate blood glucose increases and enhance plasma insulin responses. *P < 0.05, **P < 0.01 vs. control. *n* = 8–10. (**G**,**H**) The AUC of blood glucose for 120 min during GTT was lower and the AUC of plasma insulin increase for 30 min was larger in GHSR-null mice (Null) than those in wild-type mice (Wild), and comparable to those in wild-type mice receiving ghrelin antagonist [D-Lys^3^]-GHRP-6. The AUC of blood glucose rise and AUC of plasma insulin response in GHSR-null/Ins-Cre mice (Null/Cre) were indistinguishable from those in wild-type mice. In GHSR-null/Ins-Cre mice, administration of [D-Lys^3^]-GHRP-6 significantly decreased AUCs of blood glucose rise and elevated plasma insulin response. *n* = 7–8. *P < 0.05; **P < 0.01.

To assess the role of endogenous ghrelin, the effects of ghrelin antagonist on systemic glucose and insulin levels were studied in mice fasted overnight. In GTT, when a ghrelin antagonist [D-Lys^3^]-GHRP-6 (1 μmol/kg) was intraperitoneally (i.p.) injected simultaneously with 2 g/kg glucose into the wild-type mice, increases in blood glucose at 30 and 60 min were significantly attenuated (Fig. 6A) and the plasma insulin response at 15 min was markedly enhanced (Fig. 6B). Administration of another ghrelin antagonist JMV3002 (0.3 μmol/kg i.p.) also attenuated the blood glucose increase and enhanced the plasma insulin response in GTT (data not shown). These results revealed the physiological functions of endogenous ghrelin to increase blood glucose and to suppress insulin release in GTT. To examine whether these physiological functions of endogenous ghrelin are mediated by GHSR, GHSR-null mice were investigated. In GTT, the GHSR-null mice exhibited smaller blood glucose increases and larger plasma insulin responses than wild-type mice (Fig. 6C,D). Notably, the glucose and insulin curves in GTT in GHSR-null mice were similar to those in wild-type mice receiving ghrelin antagonist (Fig. 6C,D, open circles vs. Figure 6A,B, filled circles). The area under the curve (AUC) of blood glucose increase for 120 min during GTT in GHSR-null mice was significantly lower than that in wild-type mice and comparable to that in wild-type mice receiving ghrelin antagonist (Fig. 6G). The AUC of plasma insulin increase for 30 min in GHSR-null mice was larger than that in wild-type mice and comparable to that in wild-type mice receiving ghrelin antagonist (Fig. 6H). Moreover, a ghrelin antagonist failed to affect blood glucose and plasma insulin responses in GTT in GHSR-null mice (Fig. 6C,D). Notably, in GHSR-null/Ins-Cre mice, the glucose and insulin curves in GTT were similar to those in wild-type mice (Fig. 6E,F vs. Fig. 6A,B). Furthermore, administration of [D-Lys^3^]-GHRP-6 became competent to attenuate blood glucose increases and enhance plasma insulin responses in GTT (Fig. 6E,F). Consequently, the AUC of blood glucose rise was significantly decreased and that of plasma insulin response was significantly elevated to the levels indistinguishable from those in wild-type mice (Fig. 6G,H). Thus, replenishment of GHSR only in β-cells reproduced all the phenotypes of GTT and ghrelin effects on blood glucose and plasma insulin of wild-type mice.

**Figure 7:**
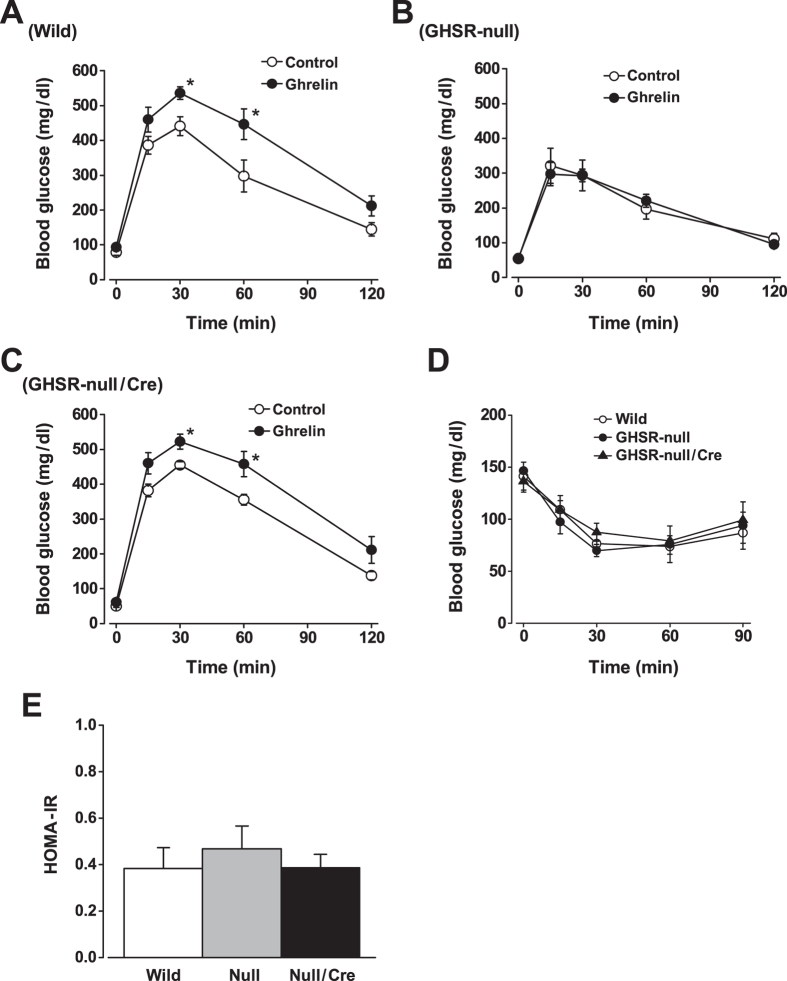
Exogenous ghrelin deteriorates glucose tolerance via β-cell GHSR. (**A**) When ghrelin at 10 nmol/kg was i.p. injected simultaneously with 2 g/kg glucose, the blood glucose levels were higher in comparison to saline-injected control values. (**B**) In GHSR-null mice, i.p. injection of ghrelin failed to affect blood glucose responses during GTT. (**C**) In GHSR-null/Ins-Cre mice, i.p. injection of ghrelin significantly elevated the blood glucose increases in GTT. (**D**) The profiles of blood glucose levels during insulin tolerance tests (ITT) and (**E**) the HOMA-IR index exhibited little differences between GHSR-null, GHSR-null/Ins-Cre and wild-type mice, suggesting that insulin sensitivity was not significantly altered. *n* = 7. *P < 0.05.

Effects of exogenous ghrelin on glucose tolerance were next examined. When ghrelin at 10 nmol/kg was i.p. injected simultaneously with 2 g/kg glucose, the blood glucose levels at 30 and 60 min were higher in comparison to saline-injected control values (Fig. 7A). In GHSR-null mice, i.p. injection of ghrelin failed to affect blood glucose responses during GTT (Fig. 7B). In GHSR-null/Ins-Cre mice, i.p. administration of ghrelin significantly elevated the blood glucose increases in GTT (Fig. 7C), indicative of restoration of the ghrelin effect on glycemia by selective re-expression of GHSR in β-cells of the GHSR-null mice. The profiles of blood glucose levels during ITT and the HOMA-IR index exhibited little differences between GHSR-null, GHSR-null/Ins-Cre and wild-type mice (Fig. 7D,E), suggesting that insulin sensitivity was not significantly altered. These results suggest that insulinostatic ghrelin function via β-cell GHSR affects systemic blood glucose levels, and β-cell GHSR is required for both endogenous and exogenous ghrelin action to regulate glucose tolerance.

## Discussion

In the present study, we found that ghrelin failed to affect glucose (8.3 mM)-induced insulin release in islets from GHSR-null mice, while ghrelin inhibited it in islets of wild-type mice. Furthermore, glucose-induced insulin release in GHSR-null islets was greater than those in wild-type islets, while insulin content per islet was unaltered in GHSR-null mice. These results in islets of GHSR-null mice are similar to those reported in ghrelin-KO mice^21^. These findings suggest that endogenous islet-derived ghrelin attenuates insulin release via GHSR. The ability of ghrelin to inhibit glucose-stimulated cAMP productions in islets and [Ca^2+^]_i_ increases in β-cells were also blunted in GHSR-null mice. These results demonstrate that ghrelin attenuates glucose-induced insulin release and cAMP and Ca^2+^ signaling via direct interaction with GHSR in β-cells.

We further demonstrated that the blood glucose-increasing effects of endogenous and exogenous ghrelin are mediated predominantly by the GHSR in islet β-cells. In the current study, ghrelin impaired and ghrelin antagonist enhanced glucose tolerance in wild-type mice, confirming our previous report^21^. In global GHSR-null mice, neither ghrelin antagonist nor ghrelin affected glucose tolerance, indicating that endogenous and exogenous ghrelin regulate glucose tolerance via GHSR. To explore the role of GHSR in β-cells, we introduced β-cell-specific re-expression of GHSR in global GHSR-null mice. In these GHSR-null/Ins-Cre mice, GHSR mRNA was re-expressed specifically in the islets, and the ability of ghrelin to inhibit insulin release, cAMP production, and [Ca^2+^]_i_ increases in islet β-cells was fully restored. In these mice in which GHSR has been rescued exclusively in β-cells, administration of [D-Lys^3^]-GHRP-6 was able to attenuate blood glucose rises and enhance plasma insulin responses in GTT. Exogenous ghrelin administration also became capable of deteriorating glucose tolerance in these mice re-expressing GHSR in β-cells. Remarkably, these *in vivo* phenotypes recaptured in GHSR-null/Ins-Cre mice were not distinguishable from those in wild-type mice. These data support that GHSR in islet β-cells primarily mediates the glycemic effect of ghrelin, at least under conditions of glucose challenge. However, our result cannot exclude a possibility that the glycemic effect of ghrelin additionally involves GHSR in other tissues implicated in insulin action^28^,^29^,^30^. Previous studies using similar Cre-mediated re-expression in the same GHSR-null line reported that the brain GHSR signaling is implicated in counter-regulatory action of ghrelin against the fasting-induced hypoglycemia^33^,^34^. Hence, GHSR in the brain might contribute to counter-regulatory action of ghrelin under hypoglycemic conditions. In addition, regulation of glucagon secretion by ghrelin via islet α-cell GHSR^35^ would be implicated under hypoglycemic conditions. Precise roles of the β-cell, α-cell and brain ghrelin/GHSR signaling in systemic glucose homeostasis remain to be further studied.

It has been well known that the GHSR is coupled to the phospholipase C-linked Gα_q/11_ family of G-proteins and [Ca^2+^]_i_ increases^2^. Our present results together with previous reports^13^,^14^ clearly demonstrate that ghrelin suppresses glucose-induced insulin release via GHSR in islet β-cells coupled to PTX-sensitive Gα_i_ and attenuation of cAMP production. Although the coupling mechanisms by which GHSR activates G_i_-proteins and suppresses cAMP cascade in β-cells are still unclear, possible direct coupling of Gα_i/o_ to GHSR has been demonstrated in *in vitro* GTPγS assays^36^,^37^. The conformation of purified monomeric GHSR was altered by ghrelin in the presence of Gα_i2_β_1_γ_2_, suggesting an interaction between GHSR and G_i_ under stimulation with ghrelin^38^. Alternatively, it has been reported that GHSR is capable of forming heterodimers with other GPCRs^39^,^40^,^41^,^42^,^43^ and thereby transduces distinct signaling. However, further studies are definitely required to elucidate the mechanisms through which the β-cell GHSR is coupled to Gα_i/o_.

Glucose-stimulated insulin secretion in β-cells is initiated by closure of the K_ATP_ channel, followed by plasma membrane depolarization. In this process, opening of background inward current through NSCCs might facilitate depolarization after K_ATP_ channel closure^32^. We previously reported that the TRPM2 channel, a type of NSCC, in β-cells plays an essential role in glucose-induced and incretin-potentiated insulin secretion^44^. Both glucose metabolism and glucagon-like peptide-1 (GLP-1) receptor stimulation increase the activity of TRPM2 channels via the cAMP signaling^32^. The present study further uncovered a novel role of TRPM2 channels in ghrelin signaling in β-cells. Ghrelin markedly counteracted the glucose (8.3 mM)-induced activation of TRPM2 current in islet β-cells. Furthermore, in islets from TRPM2-KO mice, ghrelin failed to attenuate glucose (8.3 mM)-induced insulin release. These results suggest that ghrelin suppresses glucose-induced insulin secretion at least partly by inhibiting TRPM2 channels. In contrast, noradrenaline inhibited glucose (8.3 mM)-induced insulin release in islets from TRPM2-KO mice, indicating that its insulinostatic mechanism involves the step other than TRPM2, in consistent with the consensus that noradrenaline attenuates multiple steps of insulin release including adenylate cyclase and distal exocytosis machinery^45^. TRPM2 channel activity is increased by cAMP elevating agents such as GLP-1, glucose-dependent insulinotropic polypeptide (GIP) and membrane-permeable cAMP analogues^32^. Hence, ghrelin may decrease membrane excitability by attenuating the cAMP signal-activated TRPM2 channel, thereby suppressing [Ca^2+^]_i_ signaling and insulin release. Whether other physiologic insulinostatic hormones and neurotransmitters, including somatostatin, neuropeptide Y and noradrenaline, also exert their inhibitory effects via suppression of TRPM2 currents remains to be clarified. In addition, we previously reported that ghrelin enhances voltage-dependent K^+^ (Kv) channel activity partly by suppressing cAMP pathway^13^,^14^. Possible cooperation between TRPM2 inhibition and Kv channel activation in the ghrelin action in islet β-cells remains to be elucidated.

In conclusion, the present study demonstrated that ghrelin attenuates glucose-induced insulin release via direct interaction with β-cell GHSR that is coded by ordinary Ghsr gene but exceptionally coupled to unique cAMP/TRPM2 signaling. Moreover, this interaction with β-cell GHSR largely accounts for the acute effect of ghrelin on the glycemia. Circulating ghrelin levels rise before meals and decrease after meal^46^. The reduction in plasma ghrelin level after oral glucose load is impaired in patients with type 2 diabetes^47^. The impaired ghrelin suppression after meals may partly cause impaired insulin secretion and postprandial hyperglycemia in type 2 diabetes. Plasma ghrelin levels are elevated in Chinese Han people with impaired glucose tolerance^48^. Thus, elevated ghrelin-GHSR activity could be causally implicated in type 2 diabetes. Hence, developing methods to specifically intervene the GHSR-cAMP/TRPM2 cascade in β-cells may provide a potential therapeutic tool to treat patients with type 2 diabetes.

## Methods

### Animals

Wild-type C57BL/6J mice (Japan SLC, Hamamatsu, Japan), GHSR-null mice (provided from Drs. Jeffrey M. Zigman and Joel K. Elmquist at the University of Texas Southwestern Medical Center)^6^,^49^ and TRPM2-KO mice^32^,^44^ were housed on a 12-hour light/dark cycle in accordance with our institutional guidelines and with the Japanese Physiological Society’s guidelines for animal care. KO mice were backcrossed onto a C57BL/6J mice at least for nine generations. The disrupted sequence in genomic DNA from the KO mice was detected using PCR to produce an amplicon with one primer inside the targeting sequence in combination with a gene-specific primer. Male age-matched (10 weeks-old) KO mice and wild-type littermates as controls were used. As the GHSR-null mice were generated by inserting a loxP-flanked transcriptional blocking cassette (TBC) into a Ghsr gene, mating the GHSR-null mice with tissue-specific Cre mice leads to the removal of the loxP-flanked TBC and enables the tissue-specific restoration of GHSR expression^6^,^49^. We bred the GHSR-null mice with rat insulin promoter–driven Cre recombinase (Ins-Cre) mice (The Jackson Laboratory, Bar Harbor, ME) to obtain GHSR-null/Ins-Cre mice in which GHSR is re-expressed in islet β-cells. All mice were given free access to rodent normal chow and water. Experimental protocols for animal studies were approved by the institutional committee on animal care in Jichi Medical University.

### Preparation of pancreatic islets and single β-cells

Islets of Langerhans were isolated by collagenase digestion, as reported^7^ with slight modification. Mice were anaesthetized by intraperitoneal injection of pentobarbitone at 80 mg/kg, followed by injection of collagenase at 1.14 mg/ml (Sigma-Aldrich, St. Louis, MO) into the common bile duct. Collagenase was dissolved in HEPES-added Krebs-Ringer bicarbonate buffer (HKRB) solution (in mM): NaCl 129, NaHCO_3_ 5.0, KCl 4.7, KH_2_PO_4_ 1.2, CaCl_2_ 2.0, MgSO_4_ 1.2 and HEPES 10 at pH 7.4 with NaOH, supplemented with 5.6 mM glucose and 0.1% bovine serum albumin (BSA). HKRB solution containing 0.1% BSA was used for measurements of cytosolic Ca^2+^ concentrations ([Ca^2+^]_i_) and insulin release but not for patch-clamp study. Pancreas was dissected out and incubated at 37 °C for 16 min. Islets were hand collected under a microscope and were immediately used for the measurement of insulin secretion. For β-cell experiments, islets were dispersed into single cells in Ca^2+^-free HKRB, and the single cells were plated sparsely on coverslips and maintained for 1 day at 37 ˚C in an atmosphere of 5% CO_2_ and 95% air in Eagle’s minimal essential medium containing 5.6 mM glucose supplemented with 10% fetal bovine serum, 100 μg/ml streptomycin, and 100 U/ml penicillin.

### Measurements of insulin release and cAMP productions in mouse islets

For measurements of islet insulin release, groups of 10 islets were incubated for 1 hr at 37 °C in HKRB with 5 mM glucose for stabilization, followed by test incubation for 1 hr in HKRB with 2.8 or 8.3 mM glucose. In cAMP measurements, islets were incubated for 1 hr in HKRB with 500 μM 3-Isobutyl-1-methylxanthine (IBMX), a phosphodiesterase (PDE) inhibitor (Sigma-Aldrich), to avoid degradation of cAMP in the samples. Ghrelin (Peptide Institute, Osaka, Japan) and noradrenaline (Sigma-Aldrich) was present throughout the incubation. Insulin release and total cAMP productions in islets were determined by ELISA (Morinaga, Yokohama, Japan) and EIA kit (Enzo Life Sciences, NY).

### Histological analysis of pancreatic islets

Pancreata from wild-type and GHSR-null mice were fixed in 4% paraformaldehyde, and three random sections were generated per pancreas from three mice of each genotype. The sections were incubated overnight with guinea pig anti-insulin antibody (Dako Japan, Tokyo, Japan) at a dilution of 1:1000 at 4 °C. Samples were then incubated with Alexa Fluor 488–labeled goat anti-guinea pig IgG (Molecular Probes, Eugene, OR). Immunofluorescence images were then obtained with a fluorescence microscope (Olympus, Tokyo, Japan).

### RT-PCR analysis

Total RNA of islets and pituitary was isolated using TRIzol reagent (Invitrogen, Carlsbad, CA) and treated with RQ1-DNase (Promega, Madison, WI) to remove residual contaminations with DNA. First-strand cDNA synthesis was completed using ReverTra Ace (Toyobo, Osaka, Japan) according to the manufacturer’s instructions, and then subjected to PCR amplification by HotStarTaq DNA polymerase (94 °C for 15 s, 62 °C for 30 s, and 72 °C for 30 s × 40 cycles; Qiagen, Hilden, Germany) and 2% agarose gel electrophoresis for correct product size. Primers sequence and product length were as follows: Ghsr, 5′-TATGGGTGTCGAGCGTCTT-3′ and 5′-GAGAATGGGGTTGATGGC-3′, 323 bp; and β-Actin, 5′-TTCTTTGCAGCTCCTTCGTT-3′ and 5′-CTTTTCACGGTTGGCCTTAG-3′, 402 bp.

### Measurements of [Ca^2+^]_i_ in single β-cells

Dissociated single β-cells on coverslips were mounted in an open chamber and superfused in HKRB. Cytosolic Ca^2+^ concentrations ([Ca^2+^]_i_) in single β-cells were measured at 33 °C by dual-wavelength fura-2 microfluorometry with excitation at 340/380 nm and emission at 510 nm using a cooled charge-coupled device camera^7^,^50^. The ratio image was produced on an Aquacosmos system (Hamamatsu Photonics, Hamamatsu, Japan). Data were taken exclusively from the cells which fulfilled the reported morphological and physiological criteria of β-cells including the diameter and responsiveness to glucose (8.3 mM) and K_ATP_ channel blocker tolbutamide (Tolb) (100 μM)^51^. For [Ca^2+^]_i_ measurements, β-cells were prepared from at least three mice in each experiment.

### Patch-clamp experiments in mouse single β-cells

Perforated whole-cell currents were recorded using an amplifier (Axopatch 200B; Molecular Devices, Foster, CA) in a computer using pCLAMP10.2 software, as reported^32^. For perforated whole-cell clamp experiments, pipette solution contained amphotericin B (200 μg/mL), 40 mM K_2_SO_4_, 50 mM KCl, 5 mM MgCl_2_, 0.5 mM EGTA, and 10 mM HEPES at pH 7.2 with KOH. For recording of the non-selective cation channel (NSCC) current, cells were voltage-clamped at −70 mV, which is close to potassium equilibrium potential, and treated with tolbutamide (100 μM) to inhibit the K_ATP_ channel. After recording the control current in the presence of 2.8 mM glucose, the external bath HKRB solution was changed to a solution containing 8.3 mM glucose. To examine the effects of ghrelin, cells were treated with 10 nM ghrelin 5 min before changing glucose concentrations to 8.3 mM and through the end. After measurements, the voltage-clamped β-cell was identified by insulin immunostaining^32^. Electrophysiological experiments were performed at 27 °C.

### Intraperitoneal glucose tolerance tests and insulin tolerance tests

An intraperitoneal glucose tolerance tests (GTT) and insulin tolerance tests (ITT) were performed with male GHSR-null mice and wild-type littermates, and GHSR-null/Ins-Cre mice (10 weeks-old) fasted overnight, as previously reported^21^. In GTT studies, 2 g/kg glucose was injected intraperitoneally (i.p.) into mice, followed by blood sampling from the tail vein. In ITT studies, insulin (0.75 units/kg) was i.p. injected, followed by collection of blood samples from the tail vein. Blood glucose concentrations were measured using a GlucoCard DIA meter (Arkray, Kyoto, Japan), while insulin concentrations were determined using an ELISA kit (Morinaga Institute of Biological Science). The homeostasis model assessment insulin resistance (HOMA-IR), as a surrogate index of insulin sensitivity, was calculated using the following equation: HOMA-IR = fasting glucose (mg/dL) × fasting insulin (ng/mL)/22.5.

### Statistical analysis

Data represent the means ± s.e.m. Statistical analyses were performed using the one-way ANOVA followed by Bonferroni multiple comparison tests. *P* values below 0.05 were considered statistically significant.

## Additional Information

**How to cite this article**: Kurashina, T. *et al*. The β-cell GHSR and downstream cAMP/TRPM2 signaling account for insulinostatic and glycemic effects of ghrelin. *Sci. Rep*. **5**, 14041; doi: 10.1038/srep14041 (2015).
